# Socs1-knockout in skin-resident CD4^+^ T cells in a protracted contact-allergic reaction results in an autonomous skin inflammation with features of early-stage mycosis fungoides

**DOI:** 10.1016/j.bbrep.2023.101535

**Published:** 2023-08-22

**Authors:** Yixin Luo, Maarten H. Vermeer, Sanne de Haan, Priscilla Kinderman, Frank R. de Gruijl, Thorbald van Hall, Cornelis P. Tensen

**Affiliations:** a Department of Dermatology, Leiden University Medical Center, Leiden, the Netherlands; b Department of Medical Oncology, Oncode Institute, Leiden University Medical Center, Leiden, the Netherlands; c Department of Gastroenterology and Hepatology, Leiden University Medical Center, Leiden, the Netherlands

**Keywords:** Mycosis fungoides, CD4^+^ T cells, Inflammation, Transgenic mouse, Socs1

## Abstract

Recent detailed genomic analysis of mycosis fungoides (MF) identified suppressor of cytokine signaling 1 (SOCS1), an inhibitor of JAK/STAT signaling, as one of the frequently deleted tumor suppressors in MF, and one-copy deletion of SOCS1 was confirmed in early-stage MF lesions. To better understand the functional role of SOCS1 in the genesis of MF, we used a genetically engineered mouse model emulating heterozygous SOCS1 loss in skin resident CD4^+^ T cells. In these mice an experimentally induced contact-allergic reaction was maintained for 20 weeks. Ten weeks after discontinuing contact-allergic challenges, only the skin with locally one-copy deletion of Socs1 in CD4^+^ T cells still showed high numbers of CD3^+^/CD4^+^ Socs1 k.o. cells in the dermis (p ＜ 0.0001) with prevalent Stat3 activation (p ＜0.001). And in one out of 9 mice, this had progressed to far more dramatic increases, including the thickened epidermis, and with an explosive growth of Socs1 k.o. T cells in circulation; indicative of cutaneous lymphoma. Hence, we show that Socs1 mono-allelic loss in CD4^+^ T cells locally in protractedly inflamed skin results in autonomous skin inflammation with features of early-stage MF.

## Highlights

• Mono-allelic Socs1 loss in skin-resident CD4^+^ T cells boosts skin inflammation, sustaining it with features of early-stage MF.
• Mono-allelic Socs1 loss in skin-resident CD4^+^ T cells increases activation of Stat3 in skin-inflammatory T cells.
• Mono-allelic Socs1 loss in skin-resident CD4^+^ T cells leads to pathological expansion of lymphocytes in circulation and skin.
• This transgenic mouse model opens up new possibilities of unravelling molecular mechanisms underlying the genesis of MF.

## 1 Introduction

Mycosis fungoides (MF) is characterized by the proliferation of malignant mature skin resident CD4^+^ T cells [[1], [2], [3]]. Early-stage MF often presents with limited skin lesions such as red, scaly patches or plaques and with the features of a substantial infiltration of reactive immune cells and a small group of malignant T cells [4]. In advanced stage, more infiltrated plaques, generalized erythroderma or tumors can develop with extracutaneous involvement (blood, lymph nodes, and other visceral organs) [[2], [3], [4]]. The pathogenesis of MF remains elusive. Genetic investigation of advanced MF indicates involvement of the NF-kB, TCR, and JAK/STAT pathways [5,6]. SOCS1 is a member of the suppressor of cytokine signaling (SOCS) family and inhibits immune-associated inflammatory responses mediated by the JAK/STAT pathway and controls cancer-related inflammation [7,8]. Recent research of our group has identified SOCS1 as one of the frequently deleted tumor suppressors in MF and deletions have been detected in early-stage of MF [9].

Genetically engineered mouse models (GEMMs) have been established as versatile tools to study the function of tumor suppressors and oncogenes, facilitated by the development of advanced genetic techniques. In contrast to models inoculated with patient-derived cancer cells (xenografts), GEMMs grow de novo tumors in an immune-competent natural microenvironment as an experimentally accessible model of the pathogenesis [10,11]. Thus, GEMMs can be used for the validation of candidate oncogenes, evaluation of treatment and dissection of the role of the tumor microenvironment. In mycosis fungoides, most in vivo models are xenografts and a pathogenic model of early-stage MF is lacking [12,13]. Early-stage MF skin lesions are densely infiltrated with reactive immune cells and a small group of malignant T cells. A crucial step in the progression of MF from an early, indolent stage to an advanced illness is the change in the inflammatory environment associated with the tumor [4,14]. We created and improved an autochthonous mouse model that allows selective Socs1 deletion in skin-infiltrating CD4^+^ T cells [15], based on the observation that Socs1 is one of the most prevalent genetic changes in MF. The mouse model has the CRE-loxP conditional knockout system controlled by the CD4 promoter. The deletion of Socs1 in CD4^+^ T cells can be controlled in time and location by using 4-hydroxy-tamoxifen (4OHT) to activate CRE [15].

This study is a follow-up and extension of our previous small exploratory study [15], to firm up the causal role of a SOCS1 allelic loss in the development of MF. Here, we used larger group sizes (8–9) to strengthen statistics and extended the duration of the experiment to establish more firmly what happens in the long run. A 20-week regimen of contact allergic challenges was followed by an extended 10-week observational period to study the course of skin inflammation long after discontinuing of contact allergic challenges. This 30-week experiment would also provide more time for the anticipated tumor development. Moreover, we diminished the initial systemic effects of 4-repeated hydroxy-tamoxifen applications by reducing it to just one application, which was proven to be adequate.

As chronic skin inflammation is suspected to precede MF, our experiment was targeted to answer one main question: what is the impact of allelic loss of Socs1 in a protracted skin inflammation – is it sufficient to lead up to MF? Therefore, the main experimental comparison focuses on comparing a protractedly inflamed skin with a mono-allelic loss of Socs1 in CD4^+^ T cells to one without this allelic loss. Thus, we clearly demonstrate that mice with one-copy deletion of Socs1 in skin resident CD4^+^ T cells in protractedly inflamed skin lead up to an autonomous inflammation and the features of early-stage mycosis fungoides.

## 2 Materials and methods

### 2.1 Mouse models

Socs1 fl/wt Cd4Cre+/- and Socs1 fl/wt Cd4Cre-/- were generated as presented in our previous study [15]. However, compared with the previous study, this experiment lasted more than 20 weeks longer, used a larger number of mice, and had fewer systemic effects by only using a single dose of 4OHT.

All mice were housed in individually ventilated cages, maintained under specific pathogen-free conditions, and had access to food and water ad libitum.

All mouse experiments were supervised by the animal welfare committee (IvD) of the Leiden University Medical Center and approved by the national central committee of animal experiments (CCD) under the permit number AVD116002015271, in accordance with the Dutch Act on Animal Experimentation and EU Directive 2020/63/EU.

### 2.2 Flow cytometry

Blood (50μl) was collected weekly 24 h after OXA application. Whole blood samples were processed using lysis buffer (from Hospital Pharmacy at LUMC) for 10 min at 37 °C. Cells were incubated with monoclonal antibodies for 30 min on ice.

Fluorescence-labeled antibodies included anti-mouse CD3 (clone 145-2C11, BD, The Netherlands), anti-mouse CD19 (clone 1D3, Thermo Fisher Scientific, The Netherlands), anti-mouse CD4 (clone RM4-5, Thermo Fisher Scientific, The Netherlands), anti-mouse CD8 (clone 53–6.7, Biolegend, The Netherlands) and anti-ΔhCD4 (clone RPA-T4, eBioscience™, The Netherlands). Of note, the antibody for ΔhCD4 should be specific for this fragment of human-CD4 as a reporter in the Socs1flox transgenic mouse. Samples were processed in a BD Fortessa flow cytometer and analyzed using the FlowJo software.

### 2.3 Histological and immunohistochemical analysis

All staining experiments were done on 4-μm-thick sections from formalin-fixed paraffin-embedded skin. Tissue sections were stained with hematoxylin and eosin to visualize general histological architecture. We used anti-human CD4 (1:2000, EPR6855, Abcam, The Netherlands), anti-mouse CD3 (1:200, D7A6E, Cell Signaling Technology, The Netherlands), anti-mouse CD4 (1:100, D7D2Z, Cell Signaling Technology, The Netherlands), anti-mouse CD8 (1:1600, 4SM15, eBioscience™, The Netherlands), and anti-phospho-Stat3 (1:150, D3A7, Cell Signaling Technology, The Netherlands). The scanner (3DHISTECH, Panoramic 250) was used for microscopic examination and image acquisition.

### 2.4 Immunohistochemical evaluation

The layers of the epidermis were counted within at least 5 high power fields (HPF) (20× magnification) of each slide, and the means were assessed for further statistical analysis.

The numbers of △hCD4^+^, CD3^+^, CD4^+^, CD8^+^ and phospho-Stat3 positive cells in the dermis were counted within at least 5 HPF (20× magnification) per case. The values were normalized to cells/mm2, and the mean numbers were assessed for further statistical analysis. The evaluations were conducted by two independent individuals who were blinded to the samples information.

### 2.5 Statistical analysis

A paired *t*-test was used to compare treated skin and untreated skin from the same mouse group. An analysis of covariance was used to compare the epidermis layers, CD3^+^, CD4^+^ and p-Stat3^+^ in the treated skin between two different mouse groups. A nonparametric test was used to compare CD8^+^ in the treated skin between two different mouse groups.

All statistical analyses were performed using GraphPad Prism software version 8 (GraphPad). In all cases, a P-value of 0.05 and below was considered significant (*), P < 0.01(**), P < 0.001 (***) and P < 0.0001 as highly significant.

## 3 Results

### 3.1 Experimental design

**Fig. 1:**
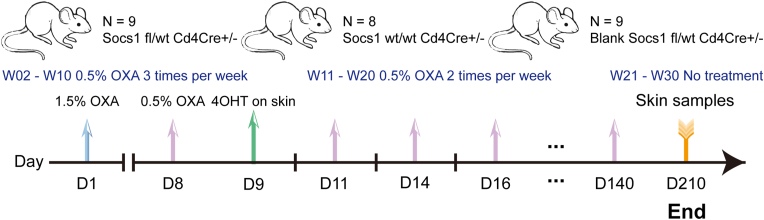
Experimental scheme employing the Socs conditional knockout mouse model. OXA: oxazolone. 4OHT: 4-hydroxy-tamoxifen. D: day. W: week. OXA application on the skin: 3 times per week from W02 to W10; twice per week from W11 to W20. Blank (Untreated) Socs1 fl/wt Cd4Cre+/- is without experimental intervention during the whole experiment. Blood (50μl) was collected weekly and was performed 24 h after OXA application. Skin samples were collected at the end of the experiment.

We used three groups of transgenic mice with both genders (the gender was not significant in the study) and with age from 7 to 9 weeks: a. an experimental group (n = 9, 4 females, 5 males) with a floxed Socs1 gene deleted through tamoxifen-activated Cre under the control of a CD4 promoter (Socs1 fl/wt Cd4Cre+/- mice, abbreviated as S+-C), b. a control group with Socs1 wt/wt Cd4Cre+/- mice (n = 8, 5 females, 3 males, abbreviated as C), and c. a blank group with Socs1 fl/wt Cd4Cre+/- mice (n = 9, 2 females, 7 males) not receiving any experimental intervention (Fig. 1).

Chronic skin inflammation was induced by maintaining a contact allergic reaction to oxazolone, as described previously [15]. To knock out Socs1, a single dose of 4OHT (20 mg/ml in ethanol, 1 mg 4OHT per mouse) was used on the shaved left flank after inducing a first contact allergic skin reaction to oxazolone, OXA (sensitizing with 1.5% concentration in acetone on the shaved abdomen and evoking with 0.5% in acetone) (Fig. 1). After that, repeated dosing of OXA (0.5% concentration) was applied on the shaved left flank three times a week from week 2 to week 10 and twice a week from week 11 to week 20, with each dose at least 48h apart (Fig. 1). The shaved right flank was only treated with a vehicle. Skin samples were collected on day 210 (Fig. 1). The shaved left flank was marked as treated skin and the right flank as untreated skin.

The mice in the blank group did not show any phenotypical deviations, and no abnormalities were seen in FACS or IHC. The data are available but not presented in the figures.

### 3.2 Single dose topical application of 4OHT is sufficient to delete Socs1 in CD4^+^ T cells

We used a single application of 4OHT on the skin to delete Socs1 by activating the CRE-loxP system. We detected the expression of truncated human CD4, (△hCD4), on cells in the blood and the skin as a reporter only expressed upon deletion of the floxed fragment in the Socs1 gene [[15], [16], [17]].

**Fig. 2:**
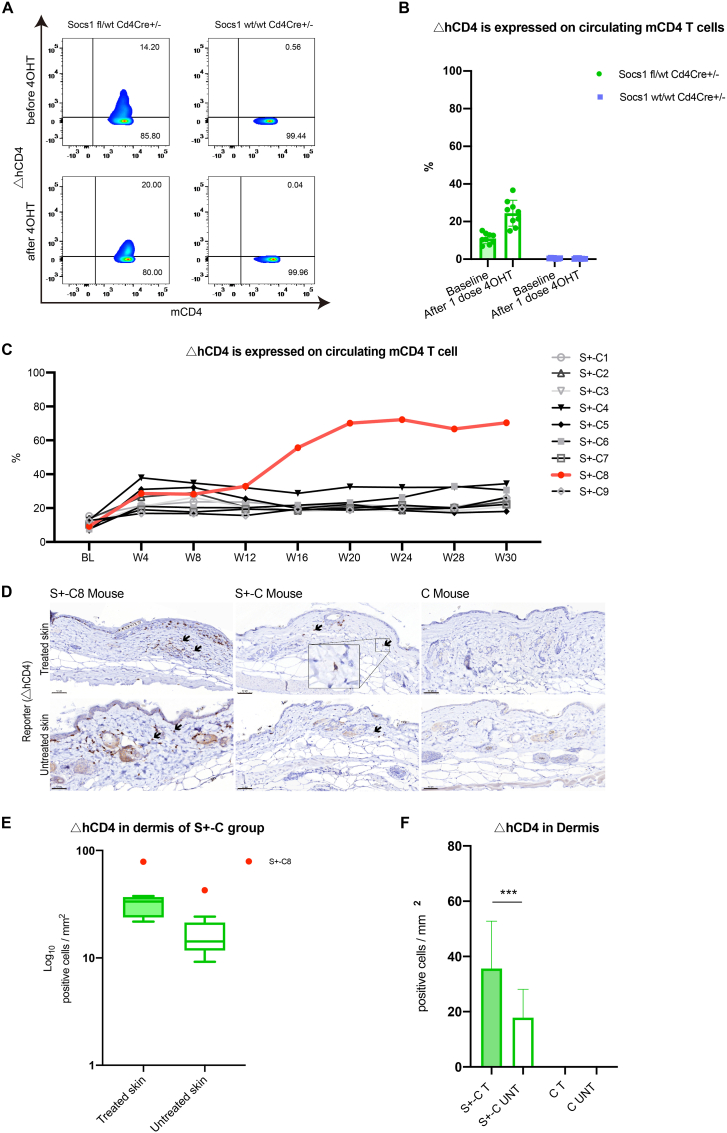
Single dose application of 4OHT knocked out Socs1 in circulating and skin-homing CD4^+^ T cells in Socs1 fl/wt Cd4Cre+/- mouse. (A). The flow cytometry results of periphery blood before and after a single dose application of 4OHT on the skin. The loss of Socs1 (measured as ΔhCD4 expression) in murine CD4^+^ T cells was analyzed. (B). The percentage of cells with loss of Socs1 (measured as ΔhCD4 expression) in total circulating murine CD4^+^ T cells in Socs1 fl/wt Cd4Cre+/- group and Socs1 wt/wt Cd4Cre+/- group before and after single dose application of 4OHT on the skin. Symbols in bar graphs represent individual mice. Data are presented as mean ± SD. ***P＜0.001. (C). The overview of the percentage of cells with loss of Socs1 deletion (measured as ΔhCD4 expression) in total circulating murine CD4^+^ T cells in Socs1 fl/wt Cd4Cre+/- group during the whole experiment. Lines represent individual mice. BL is baseline. W is week. S +-C is Socs1 fl/wt Cd4Cre+/-. (D). Immunohistochemical staining results of ΔhCD4 (to demonstrate Socs1 knockout) in the dermis from S+-C mouse and C mouse. S+-C is Socs1 fl/wt Cd4Cre+/-. C is Socs1 wt/wt Cd4Cre+/-. Scale bar: 50 μm. Black arrows: positive cells. (E). Box and Whisker plots representing the quantification of △hCD4-positive cells in the dermis of the S+-C group. S+-C is Socs1 fl/wt Cd4Cre+/-. The box in each plot spans the interquartile range of the data, with the median indicated by a horizontal line within the box. The whiskers extend to the minimum and maximum values within 1.5 times the IQR from the first and third quartiles, respectively. Outlier S+-C8 beyond this range is displayed as an individual data point. (F). Quantifying dermal ΔhCD4-positive cells in S+-C and C mice. S+-C is Socs1 fl/wt Cd4Cre+/-. C is Socs1 wt/wt Cd4Cre+/-. T is 4OHT and OXA treated skin; UNT is untreated skin. S+-C8 was excluded as an outliner. Data are presented as mean ± SD. ***P＜0.001.

A single dose of 4OHT applied topically to the skin of S+-C mice resulted in significantly increased △hCD4^+^ CD4^+^ T cells over baseline in circulation, signifying induced Socs loss in these cells, as detected by flow cytometry (Fig. 2A and B). No Socs1 knockout (△hCD4^+^) was observed in the control group (Fig. 2A). This single application importantly reduced the number of Socs1 ko cells in circulation in comparison to our previous exploratory experiment (fewer systemic effects).

The flow cytometry results showed one mouse from the experimental group, mouse S+-C8, with a strongly growing percentage of Socs1 knockout cells among the circulating CD4^+^ T cells starting from week 16. Compared to S+-C8, other mice in this group had a stable, enhanced percentage of Socs1 knockout cells in the circulating CD4^+^ T cell (Fig. 2C).

Immunohistochemical staining showed a persistent presence of Socs1 knocked out in skin resident CD4^+^ T cells on day 210 in the treated flanks of S+-C mice, as flagged by △hCD4 expression. There were no △hCD4^+^ cells in the control group mice (Fig. 2D). In the experimental group, the S+-C8 mouse was an outlier (Fig. 2E). In the dermis of S+-C mice, the quantification of hCD4^+^ cells revealed that the treated flank harbored statistically significantly more cells with Socs1 knockout cells than the untreated flank. There was no Socs1 knockout detected in the control group (Fig. 2F).

### 3.3 Persistent skin inflammation with mono-allelic Socs1 loss in CD4^+^ T cells

The immunohistopathology of skin samples from different groups of mice showed a clear effect of Socs1 knockout in CD4^+^ T cells in chronic skin inflammation. We excluded S+-C8 as an outlier when we compared the S+-C group with the C group to avoid excessive skewing by this outlier.

**Fig. 3:**
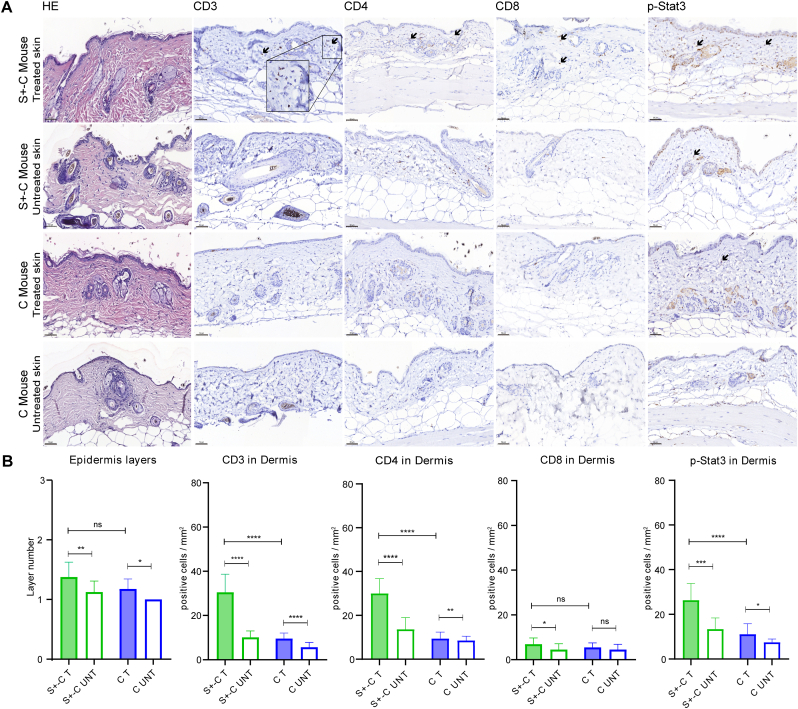
The histological and immunohistochemical results of the skin samples in different groups. (A). The HE and immunohistochemical staining for CD3, CD4, CD8, and p-Stat3 on the skin of S+-C and C group. S+-C is Socs1 fl/wt Cd4Cre+/-. C is control group. S+-C8 was excluded. Scale bar: 50 μm. Black arrows: positive cells. (B). Quantifying the epidermal layers, dermal inflammatory cells (CD3^+^, CD4^+^, and CD8^+^), and p-Stat3 positive cells in S+-C group and C group. S+-C is Socs1 fl/wt Cd4Cre+/-. S+-C8 was excluded as an outlier. C is control group. T is 4OHT and OXA treated skin; UNT is untreated skin. Data are presented as mean ± SD. *P＜0.05, **P＜0.01, ***P＜0.001, ****P＜0.0001 and ns is not significant.

The HE staining and counting of the epidermal layers demonstrated that the treated skin was thicker than the untreated skin in both the S+-C and C groups (Fig. 3A and B). There was no difference between the epidermal layers of the S+-C and C groups' treated skin (Fig. 3B).

The immunohistochemical staining of T cells showed an augmented inflammation in the skin of the S+-C group (Fig. 3A). Quantifying the staining of cells showed that in the S+-C group, the treated skin had a statistically significant increased number of CD3^+^, CD4^+^, and CD8^+^ cells in comparison with the untreated skin (Fig. 3B). In the C group, the treated skin also had a statistically significantly increased number of CD3^+^ and CD4^+^ cells compared to the untreated skin (Fig. 3B). An elevated skin inflammatory response by Socs1 deletion was demonstrated by the statistically more CD3^+^ and CD4^+^ cells in the treated dermis through the S+-C group compared to the C group (Fig. 3B).

### 3.4 More Stat3 activation in skin resident T cells after the loss of one-copy Socs1 in CD4^+^ T cells

We used immunohistochemistry to quantify the expression of p-Stat3 (activated Stat3) in the skin to determine how the absence of Socs1 in CD4^+^ T cells affected the Jak/Stat signaling pathway in mice (Fig. 3A). Quantification of these results showed that the treated skin in both the S+-C and C groups had statistically more p-Stat3^+^ cells in the dermis when compared to the corresponding untreated skin (Fig. 3B). In comparing these two groups, there was, however, a clear increase in p-Stat3^+^ cells that is statistically significant in the dermis of treated skin in the S+-C group (Fig. 3B) Hence, Socs1 knockout in cutaneous CD4^+^ T cells appears to boost Stat3 activation.

### 3.5 Pathological expansion of lymphocytes in circulation and in the skin

After 16 weeks in the experiment, one mouse, S+-C8, among the S+-C mice appeared to show explosive growth of △hCD4 expressing circulating CD4^+^ T cells, which prompted further analyses. The ratio between circulating CD3^+^ to CD19^+^ in S+-C8 decreased slightly from week 20 but increased more obviously from week 24 compared to the other mice in the same group (Fig. S1). The ratio between circulating CD4^+^ to CD8^+^ decreased more obviously from week 20 compared to the other mice in the same group (Fig. S2), indicating that there was ultimately a dominating expansion of circulating CD8^+^ cells in this mouse. Importantly, we only observed mild patches on the treated flank of this mouse (Fig. S3). There were no severe clinical skin manifestations like open wounds and ulcers (Fig. S3).

**Fig. 4:**
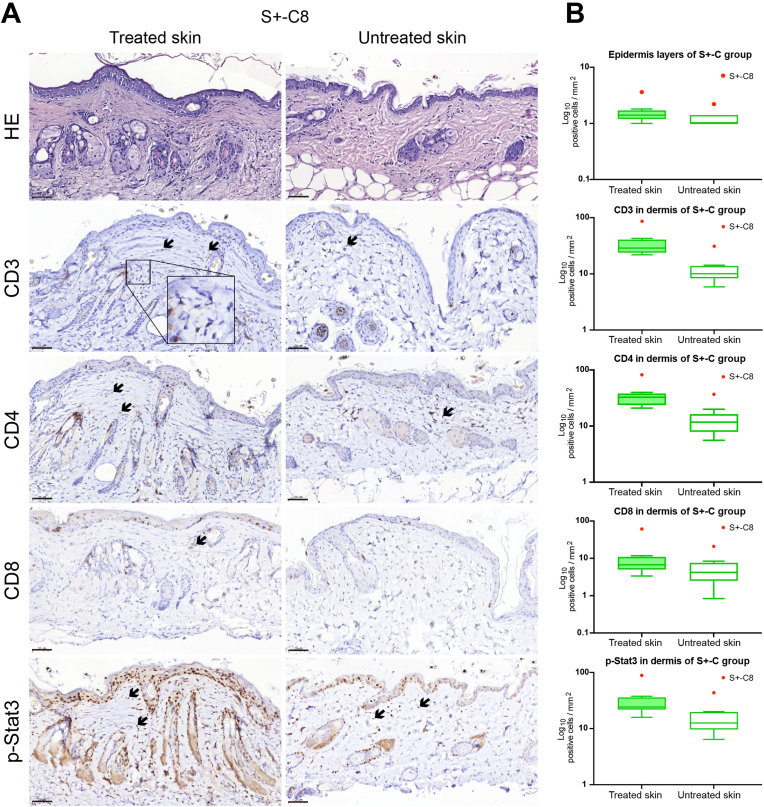
The histological and immunohistochemical results of the skins samples of S+-C8 mouse. (A). The HE and immunohistochemical staining for CD3, CD4, CD8, and p-Stat3 on the treated and untreated skin of S+-C8 mouse. S+-C is Socs1 fl/wt Cd4Cre+/-. Black arrows: positive cells. Scale bar: 50 μm. (B). Box and Whisker plots representing the epidermal layers, dermal inflammatory cells (CD3^+^, CD4^+^, and CD8^+^), and p-Stat3 positive cells in treated and untreated skin of S+-C group. S+-C is Socs1 fl/wt Cd4Cre+/-. The box in each plot spans the interquartile range (IQR) of the data, with the median indicated by a horizontal line within the box. The whiskers extend to the minimum and maximum values within 1.5 times the IQR from the first and third quartiles, respectively. Outlier S+-C8 beyond this range is displayed as an individual data point.

We also performed immunohistochemical staining and analysis of skin samples from S+-C8 (Fig. 4). CD3^+^, CD4^+^, and CD8^+^ cells infiltrating the epidermis and dermis were observed (Fig. 4). The infiltrate predominantly consisted of CD3^+^ and CD4^+^ cells (Fig. 4). The numbers of epidermal layers, inflammatory cells (CD3^+^, CD4^+^, and CD8^+^), and cells with p-Stat3 expression in the dermis of this mouse were all higher than in the remainder of the S+-C group (Fig. 4). These data demonstrated that this mouse developed a pathological expansion of immune cells in the system and skin after the Socs1 knockout in skin-resident CD4^+^ T cells.

## 4 Discussion

In MF, one-copy deletion of SOCS1 was detected in early-stage lesions and SOCS1 was found to be a tumor suppressor frequently deleted in MF [9]. Considering the grave effects caused by SOCS1 absence [18] and more particularly one-copy deletion of SOCS1 in CD4^+^ T cells in MF, the present long-term in vivo experiments focused on the impact of conditional Socs1 knockout in CD4^+^ T cells to mimic early-stage mycosis fungoides.

A single dose of 4OHT topical application was confirmed to be sufficient to knock out Socs1 in our transgenic mice. The Socs1 knockout level was stable during the whole experiment in the S+-C group (excluding S+-C8). The statistical differences between the number of inflammatory cells and Stat3 activation in treated skin of S+-C (excluding S+-C8) and treated skin of the C group show that knockout of Socs1 in skin resident CD4^+^ T cells activates Stat3 and maintains and promotes the inflammatory response. The infiltration of lymphocytes occurred mainly in the dermis. This is consistent with the results of the previous study [15]. The JAK-STAT signaling pathway is inhibited by members of the SOCS family. SOCS1 stands out as one of the most effective family members and its role is to interfere with JAK1 or JAK3, effectively reducing immune-associated inflammatory responses mediated by the JAK/STAT system. When SOCS1 is silenced, the JAK-STAT signaling pathway becomes dysregulated [8]. In lymphomas, there is evidence of DNA hypermethylation affecting the SOCS1 gene, which in turn can promote cell proliferation by enhancing JAK2 activity [19]. Studies have also reported the loss of SOCS1 in early-stage mycosis fungoides patients [9]. In 18% of MF cases, the SOCS1 promoter is inactivated due to DNA methylation [20]. Additionally, miR155, a microRNA targeting SOCS1 mRNA, has been found to be upregulated in MF cases [21]. The exacerbated inflammatory response in the skin also aligns with the fact that patients may experience more severe inflammatory lesions as mycosis fungoides disease progresses.

In the S+-C group (without S+-C8), there was a statistical difference between the number of T cells (CD3^+^, CD4^+^) and Stat3 activation in oxazolone treated versus untreated skin. Furthermore, the Socs1 knockout transgenic mice did not show other systemic abnormalities. This suggests that this mouse model produces a dense infiltrate of reactive immune cells and malignant T cells, which fits the features of early-stage mycosis fungoides [4]. This in vivo modelling preserves the microenvironment after Socs1 knockout in CD4^+^ T cells and the interaction between the oncogenic pathway and the immune system [5^,^ 12]. In the C group, the treated skin had a statistically significantly thicker epidermis, more inflammatory cells (CD3^+^ and CD4^+^), and Stat3 activation of T cells in the dermis than the untreated skin. It was evidently due to the long-term induction of chronic inflammation in the treated skin [22,23]. However, only with a loss of one-copy Socs1 in skin-resident CD4^+^ cells, in S+-C mice, the inflammatory response persisted and became more pronounced.

The most interesting observation is that under these conditions, one mouse in the experimental S+-C group showed a growing dominance of loss of Socs1 among circulating CD4^+^ T cells after week 16. The circulating CD8^+^ cells in this mouse appeared to expand strongly after week 20, perhaps in a reactive response to the prior expansion of CD4^+^ cells with Socs1 knockout. The skin samples showed a thicker epidermis with lymphocyte infiltration. The epidermis of treated and untreated skin from this mouse was thicker than other S+-C mice. The numbers of inflammatory cells CD3^+^, CD4^+^, and CD8^+^ in the dermis were also higher than in other S+-C mice. The number of CD4^+^ cells in the skin of this mouse was much higher than that of CD8^+^ cells, but in circulation, the latter ultimately dominated. The immune response toward the malignant cells, similar to the early inflammation in MF, includes a cell-mediated anti-tumor response that actively suppresses the malignant cell's expansion [[24], [25], [26]]. It also agrees with earlier findings that OXA-induced inflammation contains both CD4^+^ and CD8^+^ cells [23,27]. All the data showed a strong pathological expansion of immune cells in the skin and blood in this one mouse. Moreover, the Stat3 activation of T cells in the dermis of this mouse was also much more increased than in other S+-C mice according to the quantification of IHC staining. The constitutive Stat3 activation of T cells in the dermis and the Stat3 activation of keratinocytes in the epidermis (10 weeks after the last challenge with OXA) is also evidence of early stages of carcinogenesis and malignant progression in vivo [[28], [29], [30], [31]].

There are some limitations to the present study. The first is that only one mouse developed noticeable signs of tumorigenesis, also reflected in circulating lymphocytes. The skin showed a noticeably thicker epidermis, massive inflammatory cell infiltration and increased Stat3 activation. But the persisting skin infiltration of CD4^+^ T cells in all skin samples within the mono-allelic Socs1 in CD4^+^ T cells can be considered as a potential skin condition from which MF may develop. The observation from the one outliner mouse merely as an initial indication, suggests the need for further investigation. To establish more robust findings, it is crucial to conduct a more extensive and prolonged experiment that includes a larger sample size. Another limitation is that we were unable to include Socs1 fl/fl CD4Cre+/- mice because of a none Mendelian extremely low yield of this genotype from our breeding colony. In further research, we will perform again longer-term experiments with more mice to increase the number of mice with the features of lymphoma development.

In summary, this long-term experiment confirmed that Socs1-knockout in skin-resident CD4^+^ T cells in a protracted contact-allergic reaction results in an autonomous skin inflammation with features of early-stage mycosis fungoides.

## Ethics statement

All mouse experiments were supervised by the animal welfare committee (IvD) of the Leiden University Medical Center and approved by the national central committee of animal experiments (CCD) under the permit number AVD116002015271, in accordance with the Dutch Act on Animal Experimentation and EU Directive 2020/63/EU.

## Funding

This research was funded by the Chinese Scholarship Council.

## Author contributions

Conceptualization: CT and MV; methodology: TH, CT, FG, PK and YL; data curation: SH and YL; investigation: YL and SH; formal analysis: YL, FG and CT; writing-original draft preparation: YL; writing-review and editing: CT, FG, MV, TH, PK and SH. All authors have read and agreed to the published version of the manuscript.

## Data availability statement

The raw data supporting the conclusions of this article will be made available on request.

## Declaration of competing interest

The authors declare that they have no known competing financial interests or personal relationships that could have appeared to influence the work reported in this paper.
