# Supplementary material for: Socs1-knockout in skin-resident CD4+ T cells in a protracted contact-allergic reaction results in an autonomous skin inflammation with features of early-stage mycosis fungoides
Source: Biochem Biophys Rep. 2023 Aug 22;35:101535. doi: 10.1016/j.bbrep.2023.101535 (PMC10470183; doi:10.1016/j.bbrep.2023.101535)
Supplement: Multimedia component 1 [file mmc1.docx]

# Supplementary Figures

#
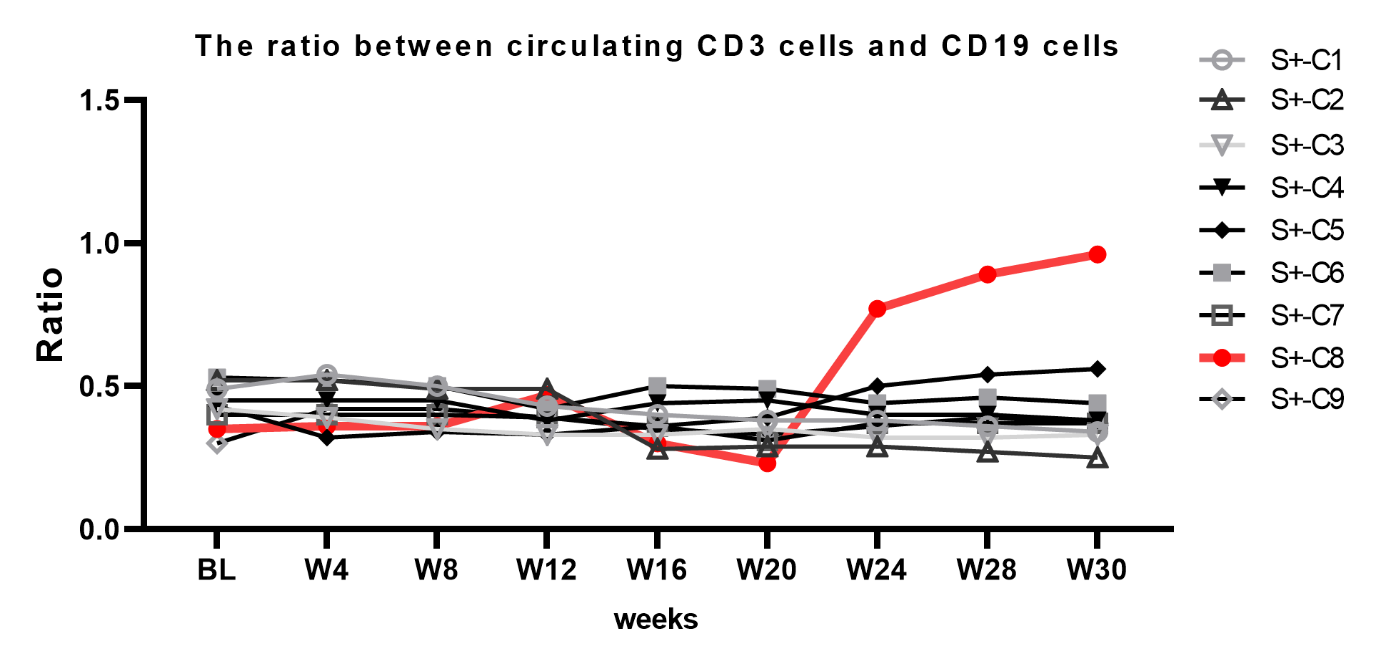


**Supplementary Figure 1.** The overview of the ratio between circulating CD3 and CD19 in Socs1 fl/wt Cd4Cre +/- group during the whole experiment. Lines represent individual mice. BL is baseline. W is week. S+-C is Socs1 fl/wt Cd4Cre +/-.


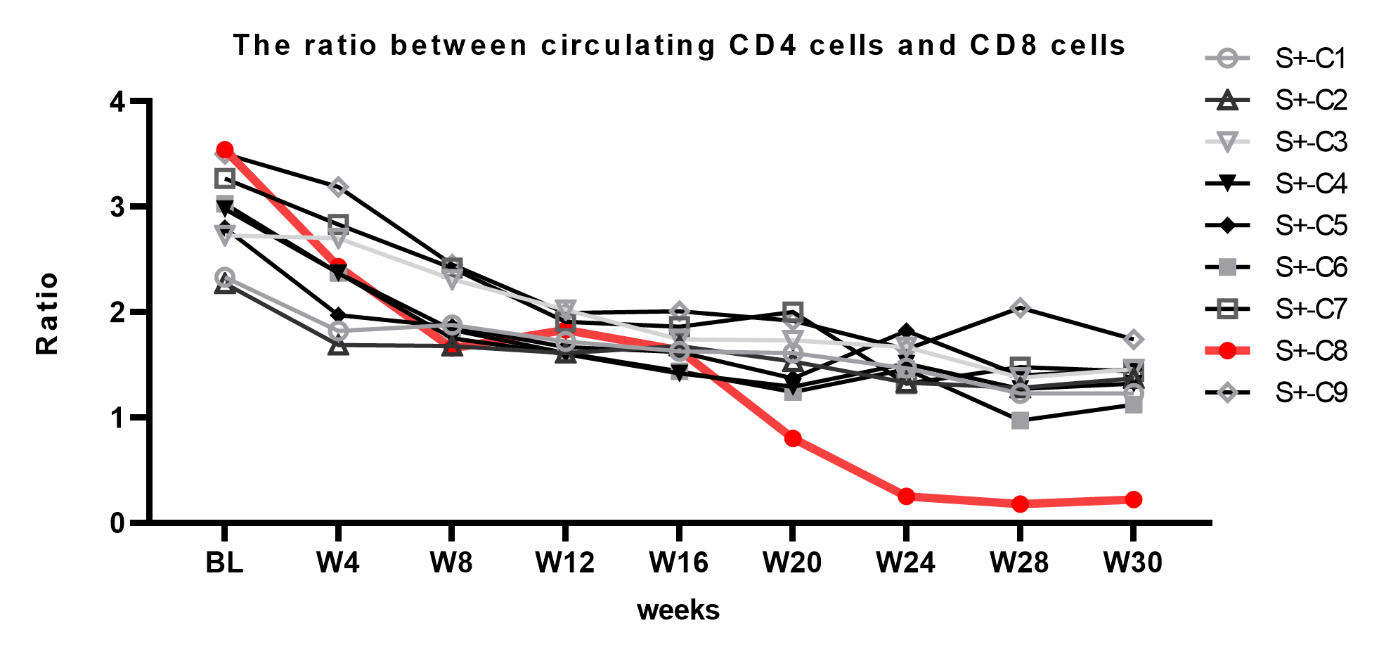


**Supplementary Figure 2.** The overview of the ratio between circulating CD4 and CD8 in Socs1 fl/wt Cd4Cre +/- group during the whole experiment. Lines represent individual mice . BL is baseline. W is week. S+-C is Socs1 fl/wt Cd4Cre +/-.


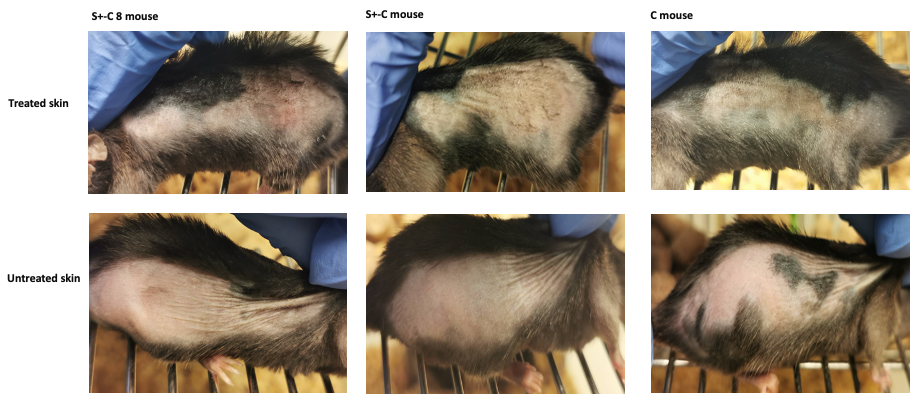


**Supplementary Figure 3.** Augmented skin inflammation induced by repeated low concentration oxazolone. Representative images of the shave treated skin and untreated skin of S--C mice, S+-C mice and C mice on day 168 (W24) during the experiment. S+-C is Socs1 -/- Cd4Cre+/- ; C is Socs1wt/wt Cd4Cre+/-.
